# Orthopedic surgical robotic systems in knee arthroplasty: a comprehensive review

**DOI:** 10.3389/fbioe.2025.1523631

**Published:** 2025-02-20

**Authors:** Xuanze Fan, Yan Wang, Shouwei Zhang, Yuan Xing, Jinhua Li, Xinlong Ma, Jianxiong Ma

**Affiliations:** ^1^ Tianjin Hospital, Tianjin University, Tianjin, China; ^2^ School of Mechanical Engineering, Tianjin University, Tianjin, China; ^3^ Tianjin Orthopedic Institute, Tianjin, China; ^4^ Institute of Medical Robotics and Intelligent Systems, Tianjin University, Tianjin, China

**Keywords:** orthopedic surgical robotic systems, robotic technology, orthopedic surgery, robotic-assisted surgery, knee arthroplasty

## Abstract

In conjunction with the accelerated evolution of robotics, the advancement of robot-assisted minimally invasive surgical systems is occurring at a similarly accelerated pace, and is becoming increasingly accepted. It is employed in numerous surgical specialties, including orthopedics, and has significantly transformed traditional surgical techniques. Among these applications, knee arthroplasty represents one of the most prevalent and efficacious procedures within the domain of robot-assisted orthopedic surgery. The implementation of surgical robotic systems has the potential to enhance the precision and accuracy of surgical outcomes, facilitate reproducibility, reduce technical variability, mitigate patient discomfort, and accelerate recovery. In this paper, a literature review of the Web of Science and PubMed databases was conducted to search for all articles on orthopedic surgical robotics through November 2024. It mainly summarizes the most commonly used and widely accepted robotic systems in the field of orthopedic surgery, with a particular focus on their application in knee arthroplasty procedures. Orthopedic robotic systems can be classified into three principal categories: autonomous robotic systems, semi-autonomous robotic systems, and teleoperated robotic systems. In the context of knee arthroplasty, the characteristics of different robotic systems are examined in relation to three types of Total Knee Arthroplasty (TKA), Unicompartmental Knee Arthroplasty (UKA) and Patellofemoral Arthroplasty (PFA). In conclusion, the current state of orthopedic surgical robotics is reviewed, and future development prospects and challenges are proposed.

## 1 Introduction

The invention of Unimate, the inaugural digitally operated and programmable robot, is regarded as the foundation of the contemporary robotics industry (Boal et al., 2024). Since that time, robotics has continued to evolve through a process of continuous exploration and innovation. As technology advanced, its applications grew beyond the traditional industrial domain to encompass the broader surgical field. In 1985, the inaugural robotic surgical system, the Puma 560, was introduced, marking a pivotal moment in history for computed tomography (CT) image-guided neurosurgical biopsies (Jacofsky and Allen, 2016; Zhang et al., 2024). This novel application introduced new concepts and methodologies to the field of surgery. In the early 1990s, Minerva was introduced to the market as a new generation of neurosurgical robots, which further advanced the development of neurosurgery (Cantivalli et al., 2023). In 1988, ROBODOC (Integrated Surgical Systems, DE, USA) emerged in the field of orthopedics. During this decade, two notable developments emerged in the field of robotic surgery: the ZEUS robotic surgical system (Computer Motion, Inc., Santa Barbara, CA, USA) and the da Vinci surgical system (Intuitive Surgical, Sunnyvale, CA, USA). Both of these systems were introduced to the market for telesurgery. Of these, the da Vinci Surgical System received Food and Drug Administration (FDA) approval for general laparoscopic surgery in 2000. This landmark event is regarded as a seminal moment in the history of surgical robotics, establishing a robust foundation for the subsequent proliferation of medical and surgical robots across diverse fields. Subsequently, medical and surgical robots have emerged in a multitude of fields, revolutionizing contemporary healthcare to an unprecedented degree.

It can be argued that orthopedics is one of the earliest surgical specialties to apply surgical robotics in clinical practice. Following over 3 decades of development, orthopedics has emerged as a highly attractive field, achieving fruitful results that are both gratifying and promising (Goldsmith, 1992; Lang et al., 2011; Banerjee et al., 2016; Christ et al., 2018). The use of surgical robots has greatly facilitated the development of total joint arthroplasty. They provide powerful robotic support for the precise preparation of bones, allowing ligaments to function as they did prior to osteoarthritic alterations. This results in a dramatic improvement in the precision of alignment reproduction and restoration of normal kinematics. Given the extensive range of potential applications for robotic surgery, prominent orthopedic companies have been incorporating these sophisticated devices into their product portfolios by developing their own proprietary systems. Consequently, the adoption of surgical robots in clinical practice is increasing as the number of approved surgical indications continues to grow and the quality of the supporting literature improves (Park and Lee, 2007; Bargar et al., 1998). Despite the fact that total joint arthroplasty usually yields excellent results, the orthopedic community is constantly striving for further innovations with the goal of increasing patient satisfaction and decreasing failure rates. The survival rate of total joint arthroplasty procedures is high, with a 10-year survival rate of up to 98% and a 20-year survival rate of up to 95% (Callaghan et al., 2009; Jauregui et al., 2015). However, the risk of failure remains, indicating that further improvements could be made. In some cases, errors in technique may result in early implantation failure, which can have a significant impact on postoperative outcomes (Jacofsky and Allen, 2016). Moreover, from a surgical standpoint, a technically proficient total joint arthroplasty may nonetheless fail to result in patient satisfaction and a state of optimal health. The reasons for this phenomenon remain unclear (Sakellariou et al., 2016; Lavand’homme and Thienpont, 2015; Liddle et al., 2015). In light of the strong desire to reduce complications and improve patient satisfaction, a number of significant technological advances have recently been made in the field of orthopedics. These advances include the use of computerized navigation, patient-specific implants, and surgical robotics (Dalton et al., 2016; Lonner and Moretti, 2016; Matassi et al., 2023). As new technologies are integrated into clinical practice, it is imperative that these advances be subjected to rigorous scrutiny for reproducibility, precision, and accuracy. Robotic systems can effectively assist surgeons in making a seamless transition from preoperative planning to intraoperative steps, which can undoubtedly enhance the accuracy and precision of surgery (Cobb et al., 2006; de Steiger et al., 2015; Fang et al., 2024; Innocenti and Bori, 2021).

The knee joint represents a crucial domain for the implementation of surgical robots in the field of orthopedics. It is evident that traditional knee surgery has a number of inherent limitations. Firstly, the preoperative preparation is frequently inadequate, which may result in a lack of comprehensiveness and precision in the development of the surgical plan. Secondly, the quality and effectiveness of the incision made during the procedure is largely dependent on the clinical experience of the surgeon. Differences in the level of surgical expertise among different surgeons may have a greater impact on the outcome of the procedure. This uncertainty not only affects the quality of prosthesis installation in the later stage but also introduces variables to the reconstruction of the patient’s lower limb force line. Furthermore, the entire knee surgery process is exceedingly arduous. In the case of Total Knee Arthroplasty (TKA), for example, the procedure requires the joint participation of many medical personnel, which not only increases the labor cost but may also lead to a variety of problems in the process of coordination and cooperation (Zhang et al., 2023). The current state of knee surgery is unable to meet the needs and requirements of the market, as it is unable to address the difficulties patients face in seeking treatment and the high costs associated with it (Gurusamy et al., 2024).

This paper is a review of the use of robotic systems in orthopedic surgery, with a particular focus on their application in knee surgery. Section 2 provides an overview of robotic systems in the field of orthopedics. Section 3 provides an overview of the techniques employed by orthopedic surgical robots in knee arthroplasty. Section 4 presents a discussion of orthopedic surgical robotics and future perspectives. Finally, Section 5 provides a brief summary.

## 2 Orthopedic surgical robotic systems

**FIGURE 1:**
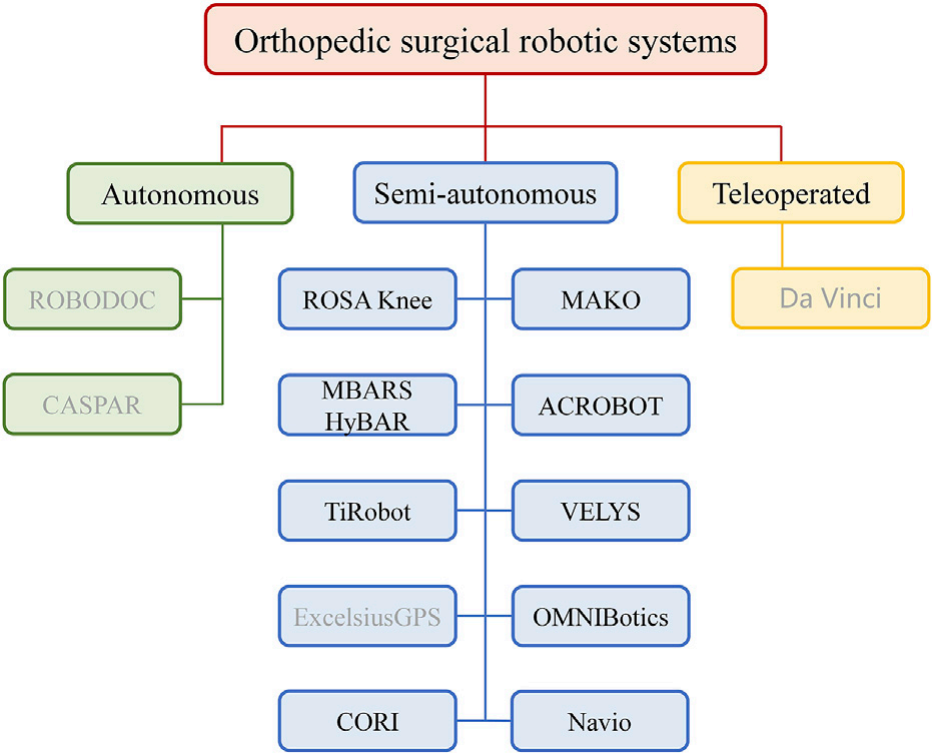
Orthopedic surgical robotic systems are categorized. The black font indicates that the robot is commonly used in knee arthroplasty surgeries, while the gray font indicates that the robot is not well suited for the procedure. Autonomous robotic systems are intelligent medical devices that can perform surgical procedures autonomously. Semi-autonomous robotic systems, also known as surgical assist robots, that require user’s physical manipulation to ensure successful surgery. Teleoperated robotic systems consists of a master robot that controls a slave robot via a network to achieve teleoperation.

Orthopedic surgical robotic systems can be classified into three principal categories according to their mode of operation: autonomous, semi-autonomous, and teleoperated robotic systems. Autonomous robotic systems are intelligent medical devices that can perform surgical procedures autonomously. It works according to a complete preoperative plan and the surgery can be performed with minimal or no intervention by the surgeon. In the event of an unexpected situation during surgery, the surgeon can halt the procedure by initiating an emergency stop. This allows for a swift and effective response to any unforeseen circumstances that may arise (Lang et al., 2011). The semi-autonomous robotic systems, also known as surgical assist robots, that require user’s physical manipulation to ensure successful surgery. In performing surgery with this type of robot, the surgeon is required to work in conjunction with the robot in order to maneuver the surgical instruments that are mounted on the robotic end-effector (EE). This process places significant demands on the robot’s human-robot interaction capabilities. Only robots with robust human-robot interaction abilities can collaborate with the surgeon effectively to ensure seamless operation (Enayati et al., 2016). A teleoperated robot, which is a master-slave robot, typically necessitates the collaboration of two robots. The master robot is operated by the surgeon in person, while the slave robot at the remote site (the surgical site) is controlled by the master robot via a network. This teleoperated approach offers a novel solution for certain complex surgical scenarios, enabling the surgeon to meticulously direct the procedure from disparate locations. Figure 1 classifies the most prevalent orthopedic surgical robots based on their utilization, with black font signifying that the robotic system is predominantly employed in knee arthroplasty, and gray indicating that the robot is not particularly well suited to the procedure.

### 2.1 Autonomous robotic systems

#### 2.1.1 ROBODOC

The ROBODOC surgical system is a fully autonomous robotic system. The system was originally designed in the 1980s for use in Total Hip Arthroplasty (THA) (Wu et al., 2023), and in 1992, it was formally introduced for patient care (Davies et al., 2007). By 2008, the FDA had granted approval for the use of ROBODOC in THA. The ROBODOC surgical system is comprised of two principal components: the ORTHODOC (3D preoperative computer modeling and planning workstation) and the ROBODOC surgical robotic arm (5-axis SACARA-type surgical robot). The system is based on preoperative CT imaging and plays a significant role in various procedures, including bone grinding and stem cell preparation for implantation. The ORTHODOC workstation is capable of generating a 3D virtual model, which can then be used to develop a customized surgical plan (Perets et al., 2020). The system performs bone motion detection and tracking by implanting a datum at a bone depth of approximately 5 mm, which allows for high tracking accuracy and resistance to interference from debris and fluids. As a fully autonomous system, ROBODOC is designed to operate without direct manual input from the surgeon. However, in the event of an unexpected situation, the surgeon is required to manually activate the emergency stop button to halt the system’s operation.

#### 2.1.2 CASPAR

**FIGURE 2:**
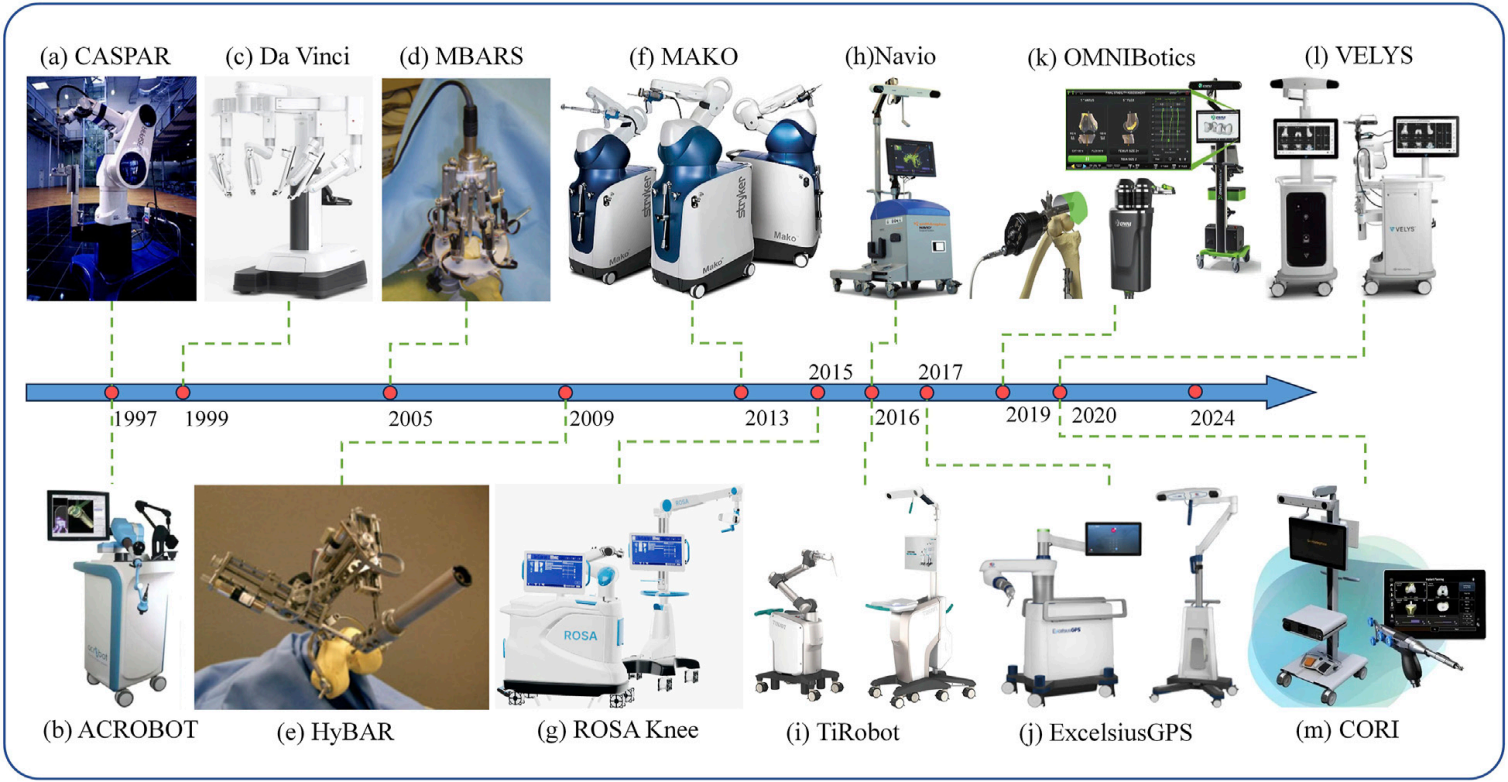
Physical drawings of various orthopedic robotic products, organized by product launch timeline. **(A)** CASPAR. **(B)** ACROBOT. **(C)** Da Vinci. **(D)** MBARS. **(E)** HyBAR. **(F)** MAKO. **(G)** ROSA Knee. **(H)** Navio. **(I)** TiRobot. **(J)** ExcelsiusGPS. **(K)** OMNIBotics. **(L)** VELYS. **(M)** CORI.

CASPAR (computer-assisted surgical planning and robotic), developed by OrthoMaquet/URS, is another autonomous 6-degree-of-freedom robotic system that has been utilized since the early stages of THA and TKA procedures, as illustrated in Figure 2A. This system shares numerous similarities with the well-known ROBODOC, which is equipped with an interactive computer system for preoperative planning based on CT images. CASPAR possesses analogous features to ROBODOC, including the capacity to automate bone milling in THA and to direct the implant into the optimal position (Ginoya et al., 2021). CASPAR exhibits analogous characteristics to ROBODOC, including the capacity to automate bone milling in THA and to direct the implant to the optimal position. Despite this, some studies have indicated that the utilisation of CASPAR markedly enhances the quality of bone preparation and the precision of implant positioning (Prymka et al., 2004; Wu et al., 2004). Nevertheless, numerous other studies have demonstrated a markedly inferior improvement in Harris hip scores following CASPAR surgery in comparison to ROBODOC. Furthermore, the procedure is considerably more time-consuming, accompanied by increased bleeding and a higher incidence of complications and the necessity for revision surgery (Siebel and Käfer, 2005). These shortcomings have led to CASPAR’s decommissioning in contemporary medical practice.

### 2.2 Semi-autonomous robotic systems

#### 2.2.1 ROSA

ROSA (Robotic Surgical Assistant) is a system that does not require either CT or fluoroscopic guidance and is primarily utilized to assist surgeons in the accurate positioning and implantation of hip components. In the course of surgical procedures, the robotic arm plays a pivotal role in aiding the guidance of surgical instruments. Fluoroscopic images obtained intraoperatively from the C-arm can be used to determine the orientation of the surgical tools in relation to the patient’s anatomy. This information can then be employed as a guide for the orientation of the bone component. The system is capable of providing measurements relative to the patient’s anatomy at three distinct time points: preoperatively, intraoperatively, and postoperatively. During the implantation of the bone component, the robotic arm remains stationary, thereby enabling the instrument to maintain a fixed orientation (Batailler et al., 2021). The ROSA system is available in two modes, either image-based or image-free. In the case of the image-based option, a preoperative three-dimensional virtual bone model is generated for the surgeon to plan the preoperative surgery. In the case of the image-free option, the patient’s skeletal anatomy is surveyed intraoperatively, and the data obtained is then used to develop an intraoperative surgical plan. Both models were found to be equally accurate in terms of resection, knee status assessment, and soft tissue assessment (Jiang et al., 2019; Rossi and Benazzo, 2023). ROSA has developed a range of orthopedic robotic systems for different body parts, including the ROSA Hip System, the ROSA Knee System, and the ROSA ONE System, where the ROSA Knee System is shown in Figure 2G.

#### 2.2.2 MAKO

The MAKO system, developed by Stryker, is an image-based system that provides haptic and auditory feedback, as illustrated in Figure 2F. Before surgery, CT images of the patient are acquired for preoperative planning. During the surgical procedure, the preoperative plan is further confirmed and adjusted based on the patient’s actual skeletal anatomy. Subsequently, the surgery is performed. The MAKO system has a distinctive feature: a haptic technology called AccuStop. This technology provides auditory beeping alerts, tactile vibration feedback, and visual feedback of color changes. This haptic technology can be employed as a protective measure for soft tissues and healthy bones during incision operations (Ginoya et al., 2021). Furthermore, the system provides haptic, visual, and audio feedback, as well as incorporating virtual fixture (VF) protection and emergency automatic shutdown as safety strategies, thus ensuring the safety of surgical procedures. Since 2021, the MAKO robotic system has been further enhanced through the integration of intraoperative sensor technology. A preoperative CT scan is initially employed to generate a 3D model, which subsequently serves as the reference for the robotic arm’s movements throughout the surgical procedure.

#### 2.2.3 Bone-attached robotic system (MBARS/HyBAR)

The MBARS (mini bone-attached robotic system) is a robot developed at Carnegie Mellon University for TKA, as illustrated in Figure 2D. This configuration employs a direct rigid mounting on the bone, which significantly diminishes the dimensions and financial outlay of the robotic system. Furthermore, the base of the bone-carrying system is affixed to the surgical site in a fixed manner, thus circumventing potential inaccuracies associated with dynamic tracking (Wolf et al., 2005). Subsequently, Shapiro et al. addressed the necessity of preoperative imaging for MBARS by developing a teleoperated haptic interface. The novel system is equipped with the capability of digitizing and describing joint surfaces within the robot’s native coordinate space.

The HyBAR (hybrid bone-attached robot) is primarily utilized for meticulous bone selection for femoral prosthesis cavity excision in patellofemoral arthroplasty, as illustrated in Figure 2E. It incorporates the advantages and disadvantages of parallel and tandem robot structures to devise a novel hybrid kinematic configuration that employs articulated prismatic joints to augment structural stiffness and introduces a novel modular clamping system to enhance the robotic program (Song et al., 2009).

In the field of orthopedics, the growing demand for minimally invasive joint arthroplasty surgery has also prompted the advancement of compact surgical robots. In comparison to robotic robots with robotic arms, small bone-mounted robots possess a more refined structure and are smaller in size, thereby reducing the space required for use and the manufacturing costs. Furthermore, they can be adapted to different patients and different types of osteotomies, thus providing greater flexibility. Consequently, the small bone-attached robotic system represents a significant area of development for orthopedic robots in the future.

#### 2.2.4 ACROBOT

The ACROBOT (active-constraint robot) system is a semi-autonomous system designed for robotic-assisted minimally invasive surgery in unicompartmental knee arthroplasty, as illustrated in Figure 2B. The mechanical structure of ACROBOT employs a 6-degree-of-freedom robot, and the control mode utilizes active constraints, which enables precise restriction of the robotic motion within a pre-defined area (Kim and Han, 2023). This enables the surgeon to perform bone cutting operations in a safe and highly accurate manner (Bargar, 2007). Moreover, the system provides haptic feedback to the surgeon, representing a prototype of modern haptic systems. It utilizes CT scans for planning before performing surgery. During the procedure, a compact ACROBOT robot is mounted on a positioning device operated by the surgeon for non-invasive anatomical marking. This enables the robot to track the drill. Upon detecting a deviation in the intended cutting area by the surgical tool, the system promptly intervenes to prevent further movement.

#### 2.2.5 TiRobot

TiRobot is the first orthopedic surgical robotic system in China to obtain a medical device registration license. It is also the first system of its kind worldwide, offering comprehensive coverage of orthopedic surgeries across three major domains: spine, joints, and trauma. This is illustrated in Figure 2I. The system is primarily utilized by orthopedic surgeons to facilitate precise surgical procedures, particularly in the domains of spine and trauma surgery. Its applications in these fields are more prevalent. The robotic system comprises a 6-degree-of-freedom robotic arm, an optical tracking system, and a main control trolley. Prior to surgical intervention, medical imaging data can be utilized by surgeons to develop a three-dimensional surgical plan. During the procedure, the robot accurately identifies the surgical site, as delineated in the plan, and provides real-time guidance to the surgeon regarding the placement and manipulation of instruments (Huang et al., 2021). The TiRobot employs an advanced optical tracking system and robotic arm technology, enabling sub-millimeter precision positioning. This is of particular importance in the context of complex orthopedic surgeries, as it has the potential to markedly enhance the precision and safety of the surgical procedure (Yang and Seon, 2023). Furthermore, the robotic system minimizes radiation exposure to the patient. In accordance with the operational protocol of the robotic system, only three imaging sessions are necessary: one prior to surgery, one following the insertion of the positioning guide pin, and one following the insertion of the screw. This significantly reduces the overall amount of X-ray radiation exposure. In the process of acquiring images, the robotic system maintains a secure and stable hold on the instruments and implants, thereby eliminating the need for medical staff to be exposed to X-rays, which effectively reduces the potential damage caused by X-rays to the medical staff. To date, the Tiangui robotic system has been utilized in over 10,000 clinical pathology procedures, demonstrating an average increase in surgical efficiency of 20% and a reduction in intraoperative radiation by approximately 70% (Nie et al., 2023).

#### 2.2.6 VELYS

The VELYS robotic system is a novel system that has been developed by Orthotaxy using proprietary technology, as illustrated in Figure 2L. The system is primarily utilized in TKA procedures. The system is equipped with high-precision sensors that are capable of sensing a variety of parameters of the surgical site in real time, including the position, shape, and hardness of the bones. Moreover, it can be utilized for specific patients in conjunction with medical imaging to facilitate precise navigation during surgical procedures. The surgeon is able to visualize the structure of the surgical site and the operation path of the robotic system on 3D images, thereby facilitating the intraoperative collection of accurate data on the bone anatomy and soft tissue envelope of the knee joint (Clatworthy, 2022). A number of clinical studies have demonstrated that the VELYS system can markedly enhance the precision and efficacy of surgical procedures. For instance, in TKA, robotic-assisted surgery has been demonstrated to reduce implant deviation and enhance joint stability and functional recovery. As technology advances, the potential applications for the VELYS orthopedic robotic system continue to grow. In addition to traditional orthopedic surgery, the robotic system can be utilized in the treatment of complex fractures, spinal surgery, and other surgical specialties (Perfetti et al., 2022).

#### 2.2.7 ExcelsiusGPS

ExcelsiusGPS is a real-time image-guided robotic system for spine surgery, as illustrated in Figure 2J. A high-resolution 3D imaging system is employed, including the fusion of multiple image modalities such as CT and MRI. The image-guidance technology provides the surgeon with clear and accurate images of the surgical area, thereby facilitating a more comprehensive understanding of the patient’s anatomy and lesions. Furthermore, the robotic guidance system of the ExcelsiusGPS system utilizes a highly rigid robotic arm, which is capable of achieving tool deflection accuracy of less than 1 mm under a lateral force of 200 N (Crawford et al., 2020). The operation of the ExcelsiusGPS robot is characterized by high reproducibility. This indicates that different surgeons can attain more uniform surgical outcomes when utilizing this robot for the same surgical procedure. This reproducibility offers the potential for standardizing surgical procedures, thereby facilitating the establishment of a more standardized surgical procedure and quality control system.

### 2.3 Teleoperated robotic systems

#### 2.3.1 Da Vinci surgical robotic system

The Da Vinci surgical robotic system was developed by Intuitive Surgical. The system is comprised of three primary components: the surgeon’s console, the patient’s surgical platform, and the image processing system. The Da Vinci robotic system is employed in a multitude of surgical specialties, including urology, gynecology, and cardiothoracic surgery, as illustrated in Figure 2C.

The Da Vinci Surgical System provides the surgeon with high-definition, three-dimensional stereoscopic vision, thereby facilitating a more detailed observation of the surgical area. The system is equipped with four robotic arms, which afford the operator seven degrees of freedom of movement. This capacity for movement is comparable to, and in some cases exceeds, the range of motion of the human wrist. This enables the surgeon to undertake more intricate procedures, such as suturing and knotting, within a confined surgical area. Moreover, the Da Vinci Surgical System is capable of supporting remote surgical operations, whereby doctors are able to control the robotic system remotely via an internet connection in order to perform surgical procedures. This provides patients in remote areas with access to high-quality medical services, and also offers a novel solution for medical assistance in emergency situations.

In the field of orthopedics, the utilization of the Da Vinci Surgical System has been predominantly constrained by the reality that the system is optimized for resilience against the rigidity of bones and is more aligned with the manipulation of soft tissue. Furthermore, the current Da Vinci robotic system is not yet capable of providing the same haptic feedback as traditional surgery, which may have implications for certain surgical procedures that require precise tactile assessment. Nevertheless, the Da Vinci system has been employed in select orthopedic procedures involving soft tissue or nerve surgery. For example, the da Vinci system has been successfully utilized for ulnar nerve decompression at the elbow joint, supraclavicular brachial plexus dissection and nerve root grafting at the shoulder joint (Beutler et al., 2013). Additionally, the da Vinci Surgical System has been utilized with success in select cases of anterior lumbar interbody fusion (ALIF) within the domain of spinal surgery (Lee et al., 2013).

## 3 Orthopedic surgical robotic systems in knee arthroplasty

### 3.1 The dilemmas of traditional orthopedic surgery

A principal application of orthopedic surgical robotic systems in the field of knee surgery is knee arthroplasty surgery. Knee arthroplasty surgery is typically employed in patients with end-stage knee osteoarthritis. In the United States, approximately 700,000 knee arthroplasty surgeries are performed annually (Swank et al., 2009). Furthermore, the number of surgeries has demonstrated an exponential growth trajectory, with total knee arthroplasty exhibiting a rate of 9.4% per year and unicompartmental knee arthroplasty demonstrating a rate of 3.2% per year (Kahlenberg et al., 2021). In terms of clinical outcomes, knee arthroplasty is a highly successful procedure, providing significant pain relief and restoring patient mobility. In the case of implants, the survival rate at 10–15 years is greater than 90% (Sharkey et al., 2014; Emmerson et al., 1996). However, traditional knee arthroplasty surgery does present certain difficulties. Firstly, with regard to surgical precision, traditional surgery depends on the surgeon’s experience and visual judgement to perform osteotomies and prosthesis installation. It is challenging to achieve the requisite degree of precision in measuring and positioning the knee. For instance, the surgeon’s experience and visual judgment may be hindered during the surgical procedure by a multitude of factors, including the lighting conditions within the operating room and the surgeon’s own level of fatigue. Such circumstances may render the osteotomy procedure and prosthesis installation exceedingly challenging. This may result in inaccurate osteotomy angles and poor prosthesis positioning, which may have a significant impact on the surgical outcome and the longevity of the prosthesis (Vogel et al., 2023). In some complex cases, such as those involving severely deformed knees, it is even more challenging to achieve accurate correction with traditional surgical methods. In such cases, there is a significant risk of residual deformity or joint instability following surgery (Parratte et al., 2010).

In regard to trauma, conventional knee arthroplasty surgery typically necessitates a substantial incision to fully expose the surgical site. Nevertheless, this methodology has the potential to inflict considerable harm to the adjacent soft tissues. In the postoperative period, patients frequently report severe pain and substantial swelling. Furthermore, the greater soft tissue damage results in a longer recovery period. The use of large incisions has been demonstrated to significantly elevate the risk of infection, in addition to the aforementioned complications (Matziolis et al., 2010). In terms of recovery time, it can be reasonably deduced that patients who have undergone conventional surgery will require a longer recovery period than those who have undergone a minimally invasive procedure. During the recovery period, patients must undergo rigorous physical therapy and functional exercises to gradually regain the mobility and functionality of the knee joint. For some patients who are older and in poorer physical condition, this undoubtedly represents a significant challenge (Ritter et al., 2011). With regard to the treatment of individual differences, it should be noted that the anatomical structure of the knee joint, the degree of lesions, and the physical condition of the patient in question vary considerably. However, traditional surgical methods tend to use relatively uniform surgical protocols and prosthesis specifications. Such an approach may result in suboptimal surgical outcomes for some patients, including prosthesis mismatch and restricted joint mobility. The use of robotic systems to assist clinicians in performing surgery can help to address these issues to some extent (Rossi et al., 2024). Although surgical robotic systems may still be in the early stages of realizing their full potential, they have demonstrated substantial advantages over traditional technologies. The utilization of surgical robotic systems has the potential to facilitate more precise surgical operations, reduce damage to surrounding soft tissues, minimize the risk of infection, expedite the recovery process, and better align with the individual needs of patients (Longstaff et al., 2009).

Knee arthroplasty can be classified according to the extent of replacement into Total Knee Arthroplasty (TKA), which involves the complete replacement of all three compartments of the knee (medial compartment, lateral compartment, and patellofemoral compartment), including the articular surfaces of the distal femur, proximal tibia, and patella (in some cases). Unicompartmental Knee Arthroplasty (UKA) is a surgical procedure that involves replacing the anterior and/or posterior cruciate ligaments and the medial and/or lateral condyles of the knee. This procedure involves replacing only the severely diseased medial or lateral compartment of the knee, while preserving structures such as the cruciate ligaments and the undamaged interphalangeal and patellofemoral articular surfaces. Bicompartmental Knee Arthroplasty (BKA) is a surgical procedure that involves replacing the surfaces of two or more compartments of the knee joint. This procedure entails the replacement of only the severely diseased medial or lateral compartment of the knee, while preserving the cruciate ligaments and undamaged structures, including the interphalangeal and patellofemoral surfaces of the knee. Patellofemoral Arthroplasty (PFA) is a surgical procedure that involves replacing the patellofemoral joint, primarily for the treatment of simple osteoarthritis of the patellofemoral joint. The following section delineates the particular applications of orthopedic surgical robotic systems in the aforementioned surgical procedures.

### 3.2 Multifaceted application of orthopedic surgical robotic system in knee arthroplasty

#### 3.2.1 TKA

The MAKO robotic arm-assisted TKA system is a semi-autonomous system that employs advanced sensors and a navigation system to track the position and movement trajectory of surgical instruments in real time, thereby providing precise guidance to the surgeon. Precise control of the osteotomy angle, position, and prosthesis size is enabled by detailed preoperative planning and real-time intraoperative navigation, thereby improving the accuracy of the procedure and the stability of the prosthesis. The utilisation of robotic systems has the potential to facilitate the attainment of precise alignment and component positional objectives as defined by surgeons, in addition to assisting in soft tissue balancing (Urish et al., 2016; Bell et al., 2016). Furthermore, it enables the creation of bespoke surgical plans based on the individual anatomy of each patient, facilitating the optimal selection of prostheses to meet the specific needs of different patients (Citak et al., 2013). In addition, the utilisation of this robotic system has the potential to reduce trauma. The use of a small incision during surgery has been demonstrated to result in reduced damage to surrounding soft tissues, decreased postoperative pain and swelling, and accelerated patient recovery. Furthermore, it safeguards vital structures, including ligaments, nerves, and blood vessels. The use of MAKO robotic assistance has been demonstrated to reduce instability and misalignment. Improved reproducibility of component positioning, alignment, and soft tissue balancing serves to eliminate alignment abnormalities and imbalances in the total knee (Bourne et al., 2010). However, the longer time required for preoperative alignment of the MAKO resulted in a longer total operative time. Some are concerned about whether the longer operative time will result in a longer postoperative hospital stay. One study compared the outcomes and length of hospital stay after 42 cases of MAKO-assisted TKA with 42 cases of conventional TKA. The results showed that the required postoperative hospital stay was essentially the same for both procedures, at approximately 1 day. Although MAKO has a relatively longer intraoperative time, its postoperative ROM recovery is significantly better than that of conventional TKA (Ng et al., 2024).

The OMNIBotics knee system has exhibited excellent performance and distinctive value in TKA. The system comprises a miniature robotic cutting guide and an active spacer, as illustrated in Figure 2K. The multi-degree-of-freedom robotic arm is capable of unrestricted movement in complex surgical environments and is able to reach the surgical site for precise maneuvers, irrespective of the knee anatomy and lesions present. The active spacer plays a pivotal role in enabling precise and reproducible tensioning of the soft tissues surrounding the knee, both before and after femoral resection. This allows surgeons to plan the resection in conjunction with the predicted ligament tension, as evidenced in previous studies (Koulalis et al., 2011; Shalhoub et al., 2018). Additionally, intraoperatively, the prosthesis-bone interface can be adjusted using the ART application software, and the depth and angle of the anterior and posterior femoral resection can be varied in small increments of 0.25 mm, thereby greatly enhancing surgical flexibility. The system is capable of adapting to the variability of bone quality observed in different patients. In the case of patients with low bone density, for instance, it can be precisely adjusted to ensure the stability of the prosthesis. Additionally, the system can accommodate the surgeon’s specific preference for the type of prosthesis fixation, facilitating a perfect fit during the endosseous trial or implant fit stage (Raj et al., 2023). Secondly, the intelligent safety system is a significant feature, comprising multiple sensors and algorithms that monitor potential safety risks during the surgical process in real time, thereby providing a robust assurance of surgical safety (DeClaire et al., 2024). Shatrov et al. (2021) performed 766 TKA using OMNIBotics since 2014, with a survival rate of 99.48% at 6-year follow-up. And also reported a survival analysis study of machine-assisted human TKA using OMNIBotics, which reported a 3-year survival rate of 99.26%. This also demonstrates that OMNIBotics is an accurate and consistent delivery tool in TKA surgery, with advantages over instrumentation, navigation aids and patient-specific cutting guides.

The ROSA Knee, a robotic system designed for robotic-assisted semi-autonomous surgery, plays a significant role in TKA. The ROSA Knee is capable of providing continuous data analysis, encompassing 3D model integration, intraoperative bone surface mapping and landmark registration, and soft tissue relaxation measurements. This significantly augments the surgeon’s capacity to position surgical instruments, perform bone resection, and evaluate soft tissue envelope balance during TKA surgery. Secondly, a high degree of automation is also a significant attribute. The system is capable of automating certain fundamental surgical procedures, thereby alleviating the surgeon’s operational burden while simultaneously enhancing the consistency and stability of the surgical process. To illustrate, during bone resection, the automated steps can guarantee that the precision and angle of each resection remain highly consistent (Thongpulsawad et al., 2024). A review of the statistical clinical data in the literature indicates that 30 TKAs performed with the ROSA Knee robot demonstrated extremely high tibial and femoral resection orientation and alignment accuracy, with a reduced number of outliers compared to traditional manual techniques or computer-guided historical controls. For instance, in the management of limb hip-knee alignment, 99.9% of limb hip-knee angulations were within the planned ±3-degree range when TKA was performed with the ROSA Knee robot, in comparison to 87.2% with conventional computer navigation and only 69.9% with manual instruments (Hetaimish et al., 2012; Parratte et al., 2023). Studies have also evaluated the accuracy of bone resection in 75 cases of TKA using the ROSA system, comparing planned versus measured angles of the distal femur, proximal tibia, and final coronal alignment. The results showed a mean difference of less than 1 mm or less than 1° (Rossi et al., 2023).

#### 3.2.2 UKA

The initial robotic system to explore UKA was the ACROBOT system, which was referenced in Section 2.2.4. In the year 2000, the ACROBOT system conducted the inaugural UKA surgery. The system features are not repeated here.

The Navio system, as a handheld, image-free robotic sculpting tool, demonstrates unique advantages and value in UKA, as illustrated in Figure 2H. The Navio system is a lightweight robotic tool whose most significant feature is that it does not necessitate in-image registration, planning, and navigation. As a semi-autonomous system, it is capable of effectively enhancing the surgeon’s movements during assisted surgical procedures, while incorporating appropriate safeguards to optimize the accuracy of the procedure. For instance, throughout the surgical procedure, the surgeon can readily grasp the tool to execute precise bone carving, while the system monitors the surgical movements in real time and alerts the surgeon in the event of potential deviations, thereby ensuring the accuracy of the surgery. Navio technology effectively optimizes the alignment of the components and ensures the precise fitting of the prosthesis, thereby increasing the success rate of the surgery. Furthermore, it preserves the volume of the osteotomy, thereby reducing the likelihood of inadvertent damage to the patient’s bone. Furthermore, the Navio system demonstrates proficiency in the restoration of joint lines, thereby facilitating the surgeon’s ability to more accurately reinstate the typical anatomy of the knee. Moreover, it is capable of quantifying soft tissue balance, thereby furnishing the surgeon with more precise soft tissue assessment data for the formulation of a more rational surgical plan (Battenberg et al., 2020). A study was conducted to assess the accuracy of bone preparation using the Navio system in 25 cadaveric specimens. The findings demonstrated that the utilisation of the Navio robotic system resulted in a notable reduction in errors in comparison to the conventional UKA bone preparation methodology. For instance, the conventional approach may be susceptible to human error, particularly with regard to the angle or depth of bone resection. In contrast, the Navio system is designed to facilitate more accurate bone preparation, which can result in a smoother bone surface and more optimal conditions for prosthesis implantation (Lonner et al., 2015). Furthermore, the Navio system is distinguished by a reduced cost and a more compact design.

The CORI system is an advanced version of the Navio system, as illustrated in Figure 2M. It is a compact and fully mobile solution that combines a 3D intraoperative imaging system with advanced robotic sculpting tools. It is much more compact and has a shorter setup time than other robotic systems such as Mako and ROSA. And compared to the NAVIO Surgical System, CORI is so portable that it can be moved from one operating room to another, resulting in improved room turnover and increased OR and room turnover. In addition, it is equipped with an intelligent mechanical bur that enables sub-millimeter precision osteotomies, helping to improve clinical osteotomy accuracy, accelerate patient recovery and improve prosthesis survival rates. The CORI Surgical System is currently considered one of the smallest, most portable and affordable robotic systems on the market. Adamska et al. (2023) statistically evaluated the postoperative outcomes of 76 Navio-assisted TKA, 71 CORI-assisted UKA and 68 manual UKA. The results showed that although robotic-assisted UKA required longer operative time compared to manual UKA, with CORI requiring less time than Navio, Navio and CORI were able to improve implant localization as well as osteotomy precision in both UKA and TKA.

#### 3.2.3 PFA

Robotic-assisted PFA represents a novel and highly innovative approach to the treatment of isolated patellofemoral arthritis. The accurate placement of the trolley implant in PFA is of great importance, yet it presents a significant challenge for clinicians. In the event that the carriage implant is incorrectly positioned, there is a significant risk of a number of unfavorable outcomes, including the emergence of diverse complications, the necessity for reoperation, and elevated revision rates. For example, an implant that is not properly positioned may result in restricted joint mobility, increased pain, or even affect the normal function of surrounding tissues. The robotic system is capable of performing high-precision three-dimensional scanning and modeling of the patient’s patellofemoral joint, enabling the accurate analysis of the joint’s anatomical structure and lesions. This, in turn, facilitates the provision of highly accurate planning for the placement of the carriage implant. During the procedure, the robotic system’s high-precision robotic arm is capable of precisely adhering to the planned trajectory, thereby ensuring that the implant is positioned in the optimal location with the optimal angle and depth. This precision not only enhances the success rate of surgery but also markedly diminishes the likelihood of complications and the probability of reoperation and revision (Dy et al., 2012). The primary robotic systems employed clinically for PFA are the Navio system and the MAKO system. The characteristics of these two systems have been previously described and will not be reiterated here. Table 1 provides a summary of the fundamental characteristics of each orthopedic surgical robotic system with applications in knee arthroplasty.

**TABLE 1:** Summary of orthopedic surgical robotic systems in knee arthroplasty applications.

Robotic systems	Developer	Applicable surgical procedures	Features	Advantage	Disadvantage
ROSA Knee	Zimmer Biomet	TKA	Provides intraoperative 3D planning and real-time data without image guidance	Real-time intraoperative landmark data acquisition	Requires additional clamping
MAKO	Stryker Surgical	TKA UKA PFA	Provides 3D planning, real-time monitoring and haptic feedback	Provides haptic feedback	Many alignment points, preoperative reliance on CT
ACROBOT	Acrobot	TKA UKA	Provides tactile feedback and active restraint	Active safety restraint system	Preoperative reliance on CT, presence of radiation exposure
TiRobot	TINAVI Medical Technologies	TKA	Sub-millimeter optical tracking and better radiation shielding	High accuracy and radiation protection	No haptic feedback
VELYS	Johnson	TKA	Provides image-free guidance and patient-specific TKA surgeries	Provides patient-specific surgeries	No haptic feedback
OMNIBotics	Corin Group	TKA	Cutting with mini robots, providing image-free guidance and intelligent safety systems	Active spacers help improve surgical outcomes	Relatively complex setup for bone-attached robot
Navio	Smith and Nephew	TKA UKA PFA	Provides real-time intraoperative imaging and monitoring without preoperative CT images	No need for CT, reducing radiation and imaging time	No haptic feedback
CORI	Smith and Nephew	TKA UKA	Provides real-time imaging and monitoring during surgery, and is compact and portable	Fast setup, portable	Long learning curve

## 4 Discussion and prospects

As technology continues to advance, robotic systems and navigation techniques demonstrate considerable potential in the field of orthopedic surgery, particularly in the context of knee arthroplasty. Especially in recent years, robotic-assisted knee arthroplasty has grown exponentially (Kow et al., 2024). Orthopedic robotic systems can automate repetitive operations such as osteotomies and drilling, thereby reducing the surgeon’s operating time and labor intensity. Conversely, the meticulous operation of the robotic system can diminish the number of errors and modifications during the procedure, thereby enhancing the overall efficiency of the operation. The implementation of an orthopedic robotic system can facilitate minimally invasive surgical procedures, thereby reducing the number of incisions and the extent of soft tissue damage (Mancino et al., 2020). To illustrate, the Navio orthopedic robot, equipped with miniaturized surgical instruments and a precise navigation system, can perform surgery through smaller incisions, thereby reducing patient trauma and postoperative pain while accelerating patient recovery. Furthermore, the orthopedic robotic system facilitates comprehensive preoperative planning through advanced imaging technology, accurately considering individual patient anatomy and providing personalized plans to enhance surgical accuracy and success. Several studies have shown that the Mako system improves TKA alignment and implant localization (Illgen et al., 2017; Blyth et al., 2017). Among them, a study by Zhang et al. (2022) reported that robotic-assisted TKA had more accurate alignment compared to manual TKA in the coronal plane of the femur, with a mean of 1.31, 95% confidence interval 1.08-1.55, p < 0.00001, and in the coronal plane of the tibia, with a mean of 1.56, 95% confidence interval 1.32-1.81, p < 0.00001. Collins et al. (2022) showed that in 72 total knee arthroplasties performed with the NAVIO system, the vast majority of cases resulted in improved implant positioning in the TKA, with 93.3% achieving correction of the desired alignment within 3 degrees of neutral. A number of studies have also evaluated the accuracy of the ROSA system and found that the ROSA Knee improved implant positioning, joint line recovery, and patient-reported outcomes in TKA (Parratte et al., 2019; Hasegawa et al., 2022). The anatomy of each patient’s knee and the lesions present therein are unique, and traditional surgical techniques are inadequate for fully addressing the individualized treatment needs of each patient. The orthopedic robotic system is capable of developing a personalized surgical plan in accordance with the specific circumstances of the patient. By means of a preoperative image evaluation and 3D reconstruction, the robotic system is able to gain an accurate understanding of the patient’s bone morphology, joint gap, and other pertinent information, thereby providing the surgeon with more precise surgical guidance. For example, the ROSA Knee orthopedic robot is capable of modifying the dimensions, configuration, and placement of the prosthesis in accordance with the unique characteristics of the patient, thereby optimizing the surgical outcome (Stauffer et al., 2023).

For clinical outcomes and patient satisfaction in robotic-assisted knee arthroplasty. Improvements in the accuracy and precision of robotic systems have been associated with superior short-term patient outcomes, such as optimal implant alignment, reduced postoperative pain, and lower complication rates. A study by Loomans et al. (2023) showed that robotic-assisted TKA reduced the length of hospital stay by an average of 1 day (p < 0.001). A study by Bhowmik-Stoker et al. (2022) found that 90% of patients treated with robotic-assisted TKA returned to driving and work within 2 months. In addition, 38% of patients returned to work within 3 weeks. Marchand et al. (2023) found that patient satisfaction was high after robotic-assisted TKA compared to manual TKA, as it improved short-term pain, physical function, and overall satisfaction scores. In addition, robotic-assisted TKA patients had significantly improved r-WOMAC pain, physical function, and overall scores at 2 years postoperatively compared with the manual TKA group. Most current studies of clinical outcomes and patient satisfaction are limited to short-term surveys. However, it is important to consider studies with limited long-term data. Therefore, ongoing research and long-term follow-up studies are needed to fully understand the potential benefits and limitations.

Nevertheless, a number of challenges remain. The first challenge is the acceptance of fully autonomous robotic systems. Safety is of paramount importance to surgeons and patients alike, and robot-assisted surgery by surgeons in the ring is currently the preferred method. However, this approach hinders the advancement of autonomous systems, and it is exceedingly difficult to enhance the quality and stability of autonomous systems. Secondly, with regard to accuracy and stability, although orthopedic robots are theoretically highly accurate, in practical applications, there are still some errors and instabilities due to the fact that robots are predominantly in the form of tandem-connected robotic arms, which are less robust (Kayani et al., 2018). For instance, the transmission mechanism of the robotic arm may exhibit gaps and signs of wear and tear, which could result in a reduction in accuracy. Additionally, the sensors may be susceptible to external interference, potentially impacting the precision of measurements. From the perspective of cost and price, the current orthopedic robotic system is bulky and heavy, necessitating the occupation of a considerable amount of operating room space. Consequently, from the standpoint of spatial utilization, its operating cost is relatively high. Furthermore, orthopedic robots lack a certain degree of flexibility. They primarily operate according to pre-set programs and parameters during surgery. In some complex surgical situations, however, surgeons may need to make adjustments and decisions based on the actual situation. In such cases, the robot may not be able to respond in a timely manner. For instance, in the event of encountering anomalies in the patient’s anatomy or the emergence of unforeseen circumstances during surgery, the robotic system may lack the capacity to make judgments and respond in a timely manner, as would a human surgeon (Hahn and Park, 2021).

In addition robotic-assisted knee arthroplasty currently has certain complications. First, although robotic-assisted knee arthroplasty improves surgical precision through advanced technology, there are still some complication problems inherent in artificial joint replacement surgery, such as periprosthetic infection, deep vein thrombosis (DVT) or pulmonary embolism (PE), prosthesis malposition, neurovascular injury, and so on. Second, some adverse events may occur that are specific to robotic-assisted knee arthroplasty. The surgical procedure for robotic-assisted knee arthroplasty includes preoperative planning, robot calibration, bone surface registration, bone cutting and grinding, and prosthesis implantation. Problems with any of the equipment aspects of the robotic system may result in forced termination of the procedure (Qin et al., 2021). In addition, complications related to the tracer fixation pins have been reported. Current robotic-assisted knee arthroplasty procedures require the temporary placement of additional fixation pins in the femur and tibia to secure the tracer in order to achieve real-time intraoperative bone tracking. Specific problems such as pin loosening, pin displacement, postoperative pin site infection, and even fracture have been reported. A loose, dislodged, or broken fixation pin can force termination of robotic surgery in 61% of cases. The incidence of skin and soft tissue infections occurring in the postoperative fixation pin tract has also been reported, and one case of pin tract resulting in osteomyelitis has been reported in the literature (Tan et al., 2023). The overall incidence of pin tract fractures is approximately 0.2%, with a predominance of lateral femoral fractures. The location of the fixation pin tract, its diameter (greater than 4 mm), fixation in the diaphysis, multiple pin placements, and the use of non-self-drilling, self-tapping pins are generally considered to be common risk factors for tract-related fractures (Haase et al., 2023).

It is anticipated that future orthopedic robotic systems will exhibit a greater degree of mechanical refinement, with a reduction in the number of smaller robots and a corresponding decrease in the space required for their operation, as well as a reduction in manufacturing costs. Moreover, it can be adapted to different patients and different types of knee arthroplasty, thereby allowing for greater flexibility. This technology has the dual benefit of reducing surgical trauma and improving surgical flexibility and adaptability, thereby rendering complex knee arthroplasty more feasible and safer (Ginoya et al., 2021). Furthermore, in the domain of software, such as navigation and positioning, surgical robotic systems have begun to integrate artificial intelligence (AI) functionality to enhance their performance, in conjunction with the advancement of AI technology. AI can assist robots in analyzing a substantial amount of data to optimize the surgical process. For instance, AI can assist surgeons in identifying lesions through image recognition technology, thereby providing the angle and position for installing prostheses in real time. Such intelligent assistance can notably diminish the burden on surgeons while enhancing the safety and efficacy of surgical procedures. Finally, the advent of 5G technology has also had a significant impact on the field of robotic-assisted surgery. There are already documented instances of successful surgical procedures conducted via teleoperation of robotic systems using 5G technology. In the future, 5G technology and robotic systems can be adapted more stably, and their high speed and low latency can ensure that the orthopedic robot can respond to commands in real time and accurately when the surgeon operates teleoperated. For example, in manipulating the robot to carry out osteotomy with millimeter precision, which can completely ensure the accuracy and safety of the surgery (Bhandari et al., 2020).

## 5 Conclusion

The results of our review showed that the number of orthopedic surgical robots has increased dramatically in recent years. This also represents an unstoppable trend for the development of orthopedic surgical robots in knee arthroplasty and even the replacement of traditional surgery. In addition, surgical robots are playing an increasingly important role in other parts and areas of orthopedics. As technology advances, robotic systems will become more intelligent and precise. In the future, robots using AI technology will be able to automatically optimize the surgical plan to improve accuracy and efficiency. And the overall equipment will gradually become more miniaturized and portable. In addition, fully autonomous robotic systems will gradually be accepted as the technology matures, and more reliable safety mechanisms will ensure surgical safety. In conclusion, the application of orthopedic robots in knee arthroplasty is an important direction of development. Although it faces challenges, the future is promising under the progress of technology and the joint efforts of all parties.
